# On the Factors Influencing Green Purchase Intention: A Meta-Analysis Approach

**DOI:** 10.3389/fpsyg.2021.644020

**Published:** 2021-04-09

**Authors:** Wencan Zhuang, Xiaoguang Luo, Muhammad Usman Riaz

**Affiliations:** School of Economics and Management, Harbin University of Science and Technology, Harbin, China

**Keywords:** green product, purchase intention, meta-analysis, cognitive factors, consumer individual characteristics, social factors

## Abstract

This study systematically analyzes the factors that affect consumers’ green purchase intention. Through a comprehensive literature review, the influencing factors of consumers’ green purchase intention are organized into three categories: cognitive factors, consumer individual characteristics, and social factors. Next, a meta-analysis of 54 empirical papers was conducted using Comprehensive Meta-Analysis 3.0 software to quantitatively assess these relationships. The results revealed that green perceived value, attitude, and green trust have a significant positive influence on green purchase intention. Perceived behavior control, perceived consumer effectiveness, and subjective norm also has a strong positive impact on green purchase intention. Collectivism has a positive effect on green purchase intention. Green perceived risk has a significant negative impact on green purchase intention. The study’s findings provide references for enterprises engaged in green product diffusion and organizations responsible for environmental protection.

## Introduction

Rapid economic development and technological progress bring even more convenience to people’s lives but also pose many challenges to the environment, such as air pollution, climate change, and global warming. These problems directly affect the sustainability of economic development, the environment, and society. It has also attracted the attention of all parties to the environment. In the past few decades, environmentally conscious consumers have achieved positive and significant growth in environmental protection activities, attitudes and knowledge. People are paying more and more attention to the environment, which directly affects the changes in personal lifestyles and values. In the case of realizing the importance of the environment, many consumers also realize that their purchasing behavior will have an impact on the ecological environment. Consumers began to change their lifestyles and business activities, and gradually tended to increase consumption of green products (Kong et al., 2014). Green products are designed to protect or improve the environment by saving energy or resources and reducing or eliminating toxic waste, pollution, and the use of toxic substances (Ottman et al., 2006). Compared with traditional products, they may be decomposable, renewable, reusable, and/or recyclable, and have little impact on the environment (Dangelico and Pontrandolfo, 2010). Green products not only pose less risk to the environment but also bring high living standards to consumers and society.

Consumers’ demand for green products has also prompted companies to pay attention to the market for green products. As sustainable development becomes a trend, the development of green products has become a broad field of social progress and commercial development, involving consumers and enterprises. As an important part of economic development, companies themselves have begun to pay attention to environmental issues. Because the development of green business helps reduce the cost of excessive useless waste, provide employees with a safe and healthy working environment, and ensure the sustainable and efficient operation of the enterprise. Therefore, companies have also begun to seek the coordinated development of environmental protection and economic growth, and strive to achieve a green economy. In order to gain a larger market for green products, companies have developed various green products to meet the needs of consumers (Dangelico and Pujari, 2010). The changes in consumers’ preferences for green products and the urgent actions needed to solve environmental problems are driving companies to seek solutions to such problems. Therefore, many companies have begun to implement green production and marketing strategies to meet customer preferences to achieve long-term business profits (Dangelico and Vocalelli, 2017; Sana, 2020).

Although consumers have more demand for green products and enterprises have a strong willingness to develop green markets, the degree of market development for green products is still insufficient. In one prior study, about 30% of consumers expressed concern about the environment and tried to translate this into their purchase behavior (Young et al., 2010). However, the green product purchase rate remains very low (Rex and Baumann, 2007). The expansion of the green product market depends on changes in consumer purchasing behavior, but research related to consumer behavior is a complex task because it involves many factors. Consumers’ green purchase intention is an important manifestation of consumers’ green behavior, and studying the factors affecting consumers’ purchase intention is of great significance for companies to formulate marketing strategies.

Consumers’ attention to the environment and green products will affect their purchase decisions (Pinto de Moura et al., 2012). To promote green products, marketers must pay attention to consumer preferences and decision-making processes (Cherrier et al., 2011). According to previous studies, there are many studies on the factors affecting consumers’ green purchase intentions (Gil and Jacob, 2018; Sun and Wang, 2019; Hashim et al., 2020; Wang et al., 2020). Lam et al. (2016) investigated a lightweight plastic bottle and verified that the product’s green perceived value had a significant positive influence on consumers’ purchase intention. Green trust has also been found to positively affect consumers’ purchase intention (Konuk et al., 2015), as have consumers’ subjective norms (Bong Ko and Jin, 2017). Sreen et al. (2018) demonstrated that consumers’ attitudes toward green purchases can affect their purchase intention and, ultimately, purchase behavior. However, other studies have shown conflicting results. Regarding consumption cognition, some scholars believe that green perceived value does not significantly affect green purchase intention (Djakasaputra et al., 2020). Other noteworthy findings are that environmental knowledge did not promote green purchase behavior (Jaiswal and Kant, 2018), subjective norms did not directly affect consumers’ green purchasing (Nguyen et al., 2017), and consumer attitudes did not significantly impact green purchase intention (Xu et al., 2020). Due to the controversial findings on this issue, there is a lack of comprehensive evaluation in the literature. To address this gap, this study uses meta-analysis methods to analyze the factors that affect consumers’ green purchase intentions. Meta-analysis is a suitable tool for finding reliable and general conclusions, compared with other research methods, meta-analysis can effectively overcome the influence of random factors such as relatively small sample size, measurement error and sampling error in a single study by summarizing and re-analyzing many subjects of the same study. It is possible to estimate the relationship among the research variables more accurately.

The theory of consumer behavior is the main theoretical method of marketing (Engel et al., 1995). According to this theory, this research constructs a framework that influences consumers’ green purchase intentions. On the basis of this research framework, combined with the expanded theory of planned behavior (TPB) and attention behavior context (ABC) theory, it analyzes the factors that affect consumers’ green purchase intentions. Based on the above theoretical basis, this study uses meta-analysis methods to analyze 54 empirical studies published from 2010 to 2020, systematically explore the factors that affect consumers’ intention to buy green products, and test the reliability of the empirical results. The results of the research provide a reference for companies to formulate marketing strategies and relevant departments to guide consumers to participate in environmental protection.

The rest of the study is arranged as follows. Section “Theoretical Framework” proposes hypothetical development based on the analysis of the existing literature; Section “Materials” prepares data for meta-analysis through data collection, data coding and data analysis; Section “Results” analyzes the specific process and final results of the meta-analysis; Section “Discussion” discusses the results of the meta-analysis; Section “Implications” discusses the implications of the research results; Section “Conclusion” draws conclusions and limitations of the research.

## Theoretical Framework

The theory of consumer behavior is one of the main theoretical methods of marketing. According to the theory of consumer behavior (Engel et al., 1995), there are three factors that affect consumers’ purchase decisions: psychological factors, individual characteristics and social factors. People’s behavior is dominated and controlled by their psychological activities, psychological factors mainly include demand, motivation and cognitive factors; individual characteristics mainly include interest, attitude and lifestyle; consumer behavior is also influenced by social factors, mainly including family, reference group and social class. Consumer purchase intention is a kind of embodiment of consumer purchase behavior, so green purchase intention can be explained by consumer behavior theory. According to this theory, this study divides the factors influencing consumers’ green purchase intention into three categories: cognitive factors, consumer individual characteristics and social factors.

The theory of planned behavior (TPB; Ajzen, 1991) is one of the most influential behavioral decision theories. TPB originated from the theory of reasoned action (TRA; Fishbein et al., 1977), which explains the influence of individual determinants, social surroundings and non-volitional determinants on intention (Han and Kim, 2010). In TPB framework three variables: attitude, subjective norm, and perceived behavioral control all together lead to the formation of a ‘behavioral intention’ which in turn influence the behavior. Meanwhile, the previous literature has extended the TPB model when studying consumers’ green purchase intention, adding new variables on the basis of the original variables. This study also uses attitude-behavior-context (ABC) theory to analyze the influence of consumer behavior on green purchase intentions (Guagnano et al., 1995). This theory provides a valuable framework for exploring consumer behavior (Goh and Balaji, 2016). Based on the previous literature, combine the expansion of TPB model and ABC theory, this study proposed green perceived value, green perceived quality, green perceived risk, perceived consumer effectiveness, environmental knowledge, environmental concern, green trust and collectivism as new predictor of green purchase intention. Previous studies have also predicted the relationship between these variables and green purchasing behavior when expanding the TPB model (Ritter et al., 2015; Maichum et al., 2016; Paul et al., 2016; Hsu et al., 2017; Taufique and Vaithianathan, 2018; Liao et al., 2020).

Therefore, based on the theory of consumer behavior, combined with the expansion of TPB model and ABC theory, this study puts forward a theoretical framework to influence consumers’ green purchase intention. The first category is cognitive factors, including green perceived value, green perceived quality, green perceived risk, perceived behavioral control, and perceived consumer effectiveness, environmental knowledge. The second category is consumer individual characteristics, including environmental concern, green trust, and attitude. The third category is social factors, including subjective norm and collectivism.

Purchasing intention is usually defined as a prerequisite for stimulating and pushing consumers to actually purchase products and services. Many studies examine consumers’ intentions to test their actual behavior. Chen and Chang (2012) believe that green purchase intention is the possibility of consumers wishing to purchase environmentally friendly products. Consumers are buying green products to protect or not damage the environment (Brian et al., 2001). Chan (2001) proposed that three items can be used to measure green purchase intentions, that is, consider buying green products, switch to other brands for ecological reasons, and switch to green versions of products. Green purchase intention is an important variable to measure customers’ current and future purchase decisions for green or environmentally friendly products. It also helps to estimate the green demand of consumers.

### Cognitive Factors

The TPB model provides a valuable framework for studying consumers’ green purchase intentions. The new variables that comprehensively affect these behavioral intentions will enhance the explanatory power of the TPB model. The literature on green purchase intention has been studied through cognitive antecedents of behavior. Cognitive factors in this study refer to consumers’ perception of green products, which is likely to have an important impact on green purchase intention.

Green perceived value refers to consumers’ overall appraisal of what they give for and receives from a product or service, based on their environmental desires, sustainability expectations, and green needs (Chen and Chang, 2012). More generally, perceived value refers to consumers’ overall assessment of the net benefit from products and services. Perceived value explains how consumers perceive the benefits and utility they obtain from using products and the time and money they give for this (Kim et al., 2012). Consumers are driven by value. Perceived value is an attribute related to product value perception, so it can establish a positive word-of-mouth effect and increase purchase intentions. Perceived value is crucial to marketing performance, because companies can cultivate consumers’ purchasing intentions through consumers’perceived value (Zhuang et al., 2010). Those consumers concerned with the environment will buy green products for their environmental benefits (Yaacob and Zakaria, 2011). As an important intermediate state variable in the process of consumer purchase, perceived value can serve as a signal of consumer judgment and a key antecedent of purchase intention (Mahesh, 2013). Tan and Goh (2018) contend that the higher the perceived value of green products, the stronger the consumer purchase intention.

Green perceived quality is consumers’ judgment of the environmental excellence of a brand (Chen and Chang, 2013). Perceived quality is a prerequisite for satisfaction and behavioral intention, reflecting consumers’ feelings of the relative advantage of a particular product or service. According to Zeithaml’s research results, perceived quality is described as a consumer’s judgment on the overall advantage of a product over alternative products (Zeithaml, 1988). Perceived quality is an important factor that affects consumers’ purchasing decisions (Nekmahmud and Fekete-Farkas, 2020). Wang et al. (2020) incorporate perceived quality as a new element in the TPB and analyze the intention of Chinese consumers to purchase green-certified food. Wu and Chen (2014) found that green perceived quality had a positive effect on consumers’ purchase intention for green products.

Perceived risk is the subjective prediction of loss, and consumers typically aim to minimize perceived risk. Chen and Chang (2012) define green perceived risk as to the expectation of negative environmental consequences associated with purchase behavior. Due to information asymmetry, it is difficult for consumers to fully understand the green product before purchasing, so they may have a certain risk perception of purchasing the green product. If consumers believe that the risk of purchasing a green product is high, they may not buy the product. Therefore, consumers’ perceived risks for green products are negatively related to green purchase intentions. Green perceived risk has been found to negatively impact green purchase intention and behavior (Wu et al., 2015). Perceived risk has a negative impact on consumers’ purchasing decisions, and it will affect consumers’ behavior. Accordingly, as green perceived risk falls, consumers’ green purchase intention is likely to increase (Tarabieh, 2020).

Perceived behavioral control refers to an individual’s judgment of their ability to perform a specific behavior (Ajzen, 1991). As an important part of the TPB model, perceptual behavior control is the perception of the difficulty of performing a specific behavior, that is, the degree to which the individual feels that the execution or non-execution of the behavior in question is under their voluntary control (Ajzen, 2006). Thus, it is the degree of control that one perceives over the performance of the behavior. When confronted by external factors while making purchase decisions, consumers who perceive that they have more resources and opportunities will have higher perceived behavioral control. Previous studies have shown that consumers tend to be more willing to buy green products when they think they can control these uncontrollable external factors (Xu et al., 2020). Wang X. et al. (2019) studied the influencing factors of consumer purchase intention in developing countries; they found that perceived behavioral control had a significant effect on purchase intention among Tanzanian consumers but not Kenyan consumers.

Perceived consumer effectiveness is the degree to which consumers think their individual actions contribute to solving problems—a subjective perception of the role of one’s own efforts (Ellen et al., 1991). Perceived consumer effectiveness involves a person’s belief that they themselves can contribute to solutions and reduce negative environmental impacts (Tan, 2011). Perceived consumer effectiveness has received great attention in understanding consumer behavior. In previous literature, perceived consumer effectiveness has been identified by researchers as an important factor in understanding consumers’ environmentally friendly purchasing behavior (Dagher and Itani, 2014; Benda-Prokeinová et al., 2017). For example, Sharma and Dayal (2017) found that perceived consumer effectiveness has a positive effect on green purchase intention. This factor has been found to relate directly to consumers’ attitudes toward green products and to be an important predictor of purchase intention (Sharma and Foropon, 2019).

Environmental knowledge is knowledge of the environment, key relationships that lead to environmental impacts, and environmental responsibility of the individual that leads to sustainable development (Fryxell and Lo, 2003). In the literature on environmental knowledge, researchers usually use different environmental knowledge concepts to predict individual green behavior: general or specific environmental knowledge, and subjective or objective environmental knowledge (Lee, 2017). This research defines environmental knowledge as a person’s perception of one’s own understanding of general environmental issues. The literature suggests that as consumers accumulate more environmental knowledge, their attention toward purchasing green products increases. Environmental knowledge has a significant positive impact on consumers’ intention to purchase environmentally friendly products (Wang et al., 2014). Specifically, consumer environmental knowledge has been identified as an important predictor of Ahmad and Thyagaraj (2015) and positive influencer of Choi and Johnson (2019) green purchase intention. Based on the above analysis, the following hypotheses are proposed:

H1: Green perceived value has a positive effect on green purchase intention.
H2: Green perceived quality has a positive effect on green purchase intention.
H3: Green perceived risk has a negative effect on green purchase intention.
H4: Perceived behavioral control has a positive effect on green purchase intention.
H5: Perceived consumer effectiveness has a positive effect on green purchase intention.
H6: Environmental knowledge has a positive effect on green purchase intention.

### Consumer Individual Characteristics

Because consumers are heterogeneous, there are also differences in their purchase intentions for green products. Although researchers have improved the explanatory power of TPB by adding personality constructs (Rhodes et al., 2002), there are few studies on the relationship between personality traits and eco-friendly behaviors (Dezdar, 2017).

Environmental concern is the degree of concern over environmental problems, and indicative of efforts to solve these problems (Dunlap and Jones, 2002). Environmental concern is considered to be a key environmental factor for analyzing personal characteristics of green marketing. Consumers who pay more attention to environmental problems tend to have a positive attitude toward green products, thus maintaining a healthy and green lifestyle (Paul et al., 2016). Consumer attention to the environment will affect purchase decisions, especially for green products. Nekmahmud and Fekete-Farkas (2020) studied green purchases by young consumers and found that environmental concerns had a significant impact on their decisions. Hartmann and Apaolaza-Ibáñez (2012) investigated the direct and indirect effects of environmental concerns and found a positive impact on consumer attitude toward and purchase intention for green energy brands.

Trust is considered a common mechanism for reducing perceived transaction risk by increasing expectations of positive outcomes and certainty of how trustees will behave. Chen (2010) expresses green trust as a willingness to rely on an object based on beliefs or expectations arising from its credibility, benevolence, and ability to perform in the environment. Compared with traditional products, consumers often need more trust when purchasing green products. Consumer trust is an important determinant of purchase intention. Many consumers lack an understanding of green products, which increases the influence of trust on their purchase intention. Tarabieh (2020) found that green trust significantly impacted green purchase intention.

As one of the three core concepts in the theory of planned behavior, attitude refers to the positive or negative evaluation or appraisal of a particular object (Ajzen, 1991). Attitudes may reveal consumer perceptions of the product. Previous research has focused on the relationship between attitude and behavioral intention, and believed that attitude is an indispensable variable when predicting consumers’ purchase intention. A positive attitude often has a more positive impact on behavioral intention (Arli et al., 2018). Many scholars have found that a positive attitude positively influences green purchase intention in studies of the relationship between green products and environmental behaviors (Wang et al., 2016). Moreover, a positive attitude has been found by many studies to positively impact consumer purchase intention in relation to green hotels (Chen and Tung, 2014; Teng et al., 2015) and organic food (Wang X. et al., 2019). Based on the above analysis, the following hypotheses are proposed:

H7: Environmental concern has a positive effect on green purchase intention.
H8: Green trust has a positive effect on green purchase intention.
H9: Positive attitude has a positive effect on green purchase intention.

### Social Factors

Consumer purchase intention for green products is affected by not only individual factors but also the social environment and other people. Social factors affect individual behavior decisions in many ways, such as social pressure from other people and collectivist ideas. This research mainly studies the impact on green purchase intention from two aspects: subjective norms and collectivism.

Subjective norm refers to the social pressure perceived by individuals on whether to carry out or refrain from a certain behavior (Ajzen, 1991). In decision-making, individuals are often influenced by the people around them. It reflects how individuals are affected in society, that is, if they participate in certain behaviors, how their reference group will perceive them. Previous research has shown that people comply with subjective norms because they are afraid of social pressure from primary referents, or because their referents provide them with guidance on appropriate or beneficial behaviors in society. Bong Ko and Jin (2017) investigated female college students in China and the United States; in both countries, subjective norms had a positive impact on consumers’ green purchase intention. When consumers realize that their “important others” recognize green purchase behavior, they tend to adopt it. For example, Yeon Kim and Chung (2011) found that if “important others” thought organic skincare products were good, consumers had more intention to purchase these products.

Collectivism is an important value that affects people’s decision-making and consumption behavior (Laroche et al., 2001). It holds that group interests are more important than individual needs and desires. In general, people from individualistic cultures tend to be independent and self-oriented whereas those from collectivistic cultures are more interdependent and group-oriented. Collectivism emphasizes interdependence, in-group harmony, family security, group-oriented goals, and cooperation. People with strong collectivism prioritize collective interests over individual interests and are willing to sacrifice the latter for the former (Zhao and Chen, 2008). Collectivism has been found to affect many kinds of social behavior. Collectivist people tend to be more environmentally friendly, as they typically pay more attention to how their behavior impacts on society, so when making purchase decisions they are inclined to choose green products (Kim, 2011). Considering that the final result of environmental consumption is a large-scale improvement of social well-being, collective efficacy beliefs may have a greater impact on green purchase intentions than self-efficacy. Lee (2017) found that collectivism had a significant impact on the green purchase intention of Chinese consumers. Therefore, the concept of collectivism has an important influence on consumers’ green purchase intentions. Based on the above analysis, the following hypotheses are proposed:

H10: Subjective norm has a positive effect on green purchasing intention.
H11: Collectivism has a positive effect on green purchasing intention.

**FIGURE 1:**
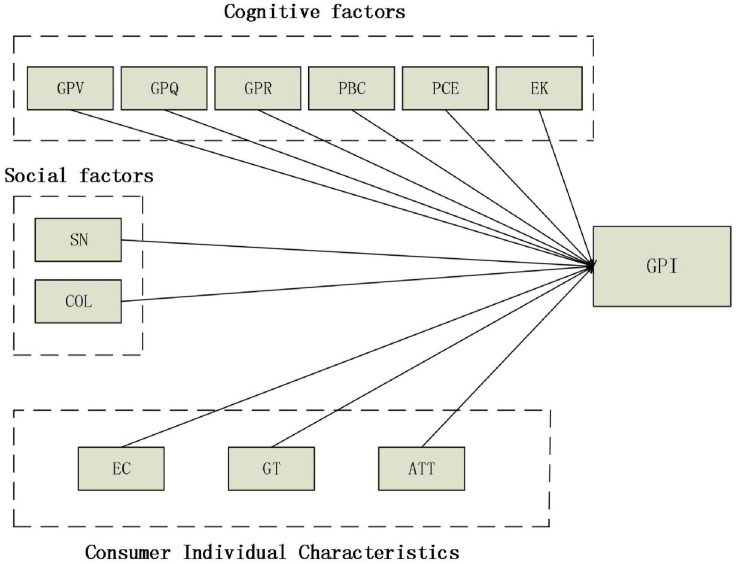
Proposed research framework. GPV, green perceived value; GPQ, green perceived quality; GPR, green perceived risk; PBC, perceived behavioral control; PCE, perceived consumer effectiveness; EK, environmental knowledge; EC, environmental concern; GT, green trust; ATT, Attitude; SN, subjective norm; COL, collectivism.

Based on the above hypotheses, the proposed research framework is depicted in Figure 1.

## Materials

### Data Collection

This study comprehensively searched the relevant literature. Works were mainly retrieved from the following databases: Springer, EBSCO, Emerald, Elsevier, CNKI, Google Scholar, and Web of Science. In terms of time frame, the search included studies published or made available online from January 2010 to October 2020. Searches used a combination of the following keywords: “green products,” “green purchase intention,” “green purchase behavior,” “green perceived value,” “green perceived quality,” “green perceived risk,” “perceived consumer effectiveness,” “perceived behavioral control,” “environmental knowledge,” “environmental concerns,” “green trust,” “attitude,” “subjective norm,” and “collectivism.”

### Selection Criteria

**FIGURE 2:**
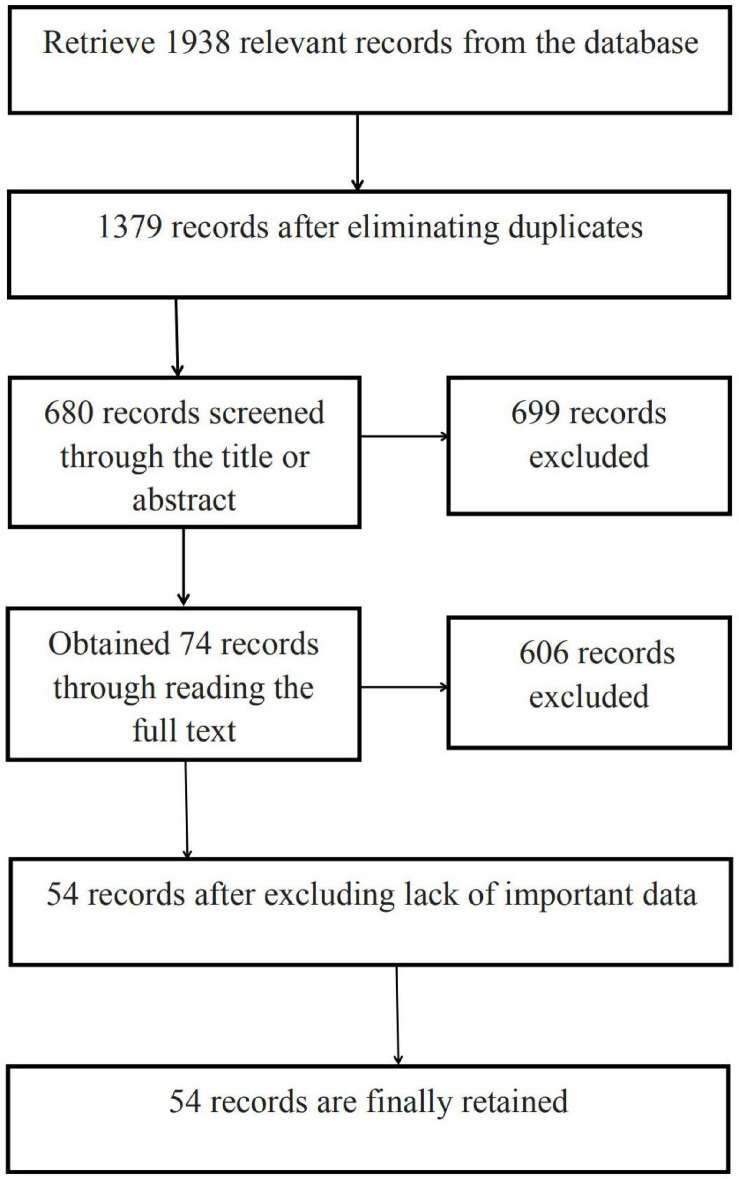
Data collection progress.

Studies were screened using the following criteria: (1) quantitative research related to green purchase intention, excluding theoretical research, reviews, etc.; (2) containing correlation coefficients or other values convertible to them, such as *t*-values and *F*-values; (3) independent samples— if multiple studies used the same sample for empirical analysis, only one was selected for the meta-analysis; (4) clear sample size; and (5) testing at least two variables or relationships of an original or modified model. According to the criteria, we search and screen studies. First, we found 1,938 studies in the database. After eliminating duplicates, 1,379 studies were identified. After scanning the title or abstract, 680 remained. Then, to ensure the accuracy of the research results, the full text of each study was screened according to the inclusion criteria, which left 74 studies. Finally, after excluding studies that lacked important evidence or did not otherwise meet the requirements of our meta-analysis, 54 studies were retained. These studies are all journal articles. The screening process is shown in Figure 2.

### Data Coding

As the data used in this research were derived from other studies differing greatly in terms of sample selection and data processing, it was necessary to code the data uniformly. To ensure the reliability of coding, the work was performed by two graduate students. The coding mainly includes two types of content: description items and the effective values of statistical items. The main description items are author, sample size, country or area, respondents, and year of publication. The main effect values of statistical items are correlation coefficients or other statistics, such as *t*-values and *F*-values, that can be converted into them. Correlation coefficients are used as the evaluation index in this meta-analysis. After each study was coded, a certain number of studies were randomly selected and coded by another researcher to ensure the accuracy of coding. Any inconsistencies in coding content were resolved through revisiting the original text and discussing until consensus was reached. The 54 studies included in the meta-analysis are listed in Table 1. The relevant data are shown in Table 2.

**TABLE 1:** Studies selected for meta-analysis.

References	Sample size	Country or area	Respondent
Sreen et al., 2018	452	India	Educated urban consumers
Karatu and Mat, 2015	440	Nigeria	College student
Tarabieh, 2020	432	Jordan	Customers who have purchasing history or perspective of such green food and beverage brands or commodities
Chen and Deng, 2016	306	China	Respondents on the online survey
Wang H. et al., 2019	236	China	Respondents on the online survey
Chen and Chang, 2012	258	Taiwan	Consumers with experience in purchasing electronic products
Wang, 2014	1866	Taiwan	Respondents on the online survey
Lee, 2017	357	Korea	Consumers crossing the mall entrance
Lee, 2017	398	China	Consumers crossing the mall entrance
Xu et al., 2020	451	China	The customers who visited the Red Star Macalline furniture store.
Paul et al., 2016	521	India	Adults on the Internet
Ahmad and Thyagaraj, 2015	270	India	Respondents who had the purchase experience of electronic products.
Kabaday et al., 2015	172	Turkey	College student
Jaiswal and Kant, 2018	351	India	Young adult and educated segment of consumers (age 18 or above)
Ali and Ahmad, 2012	377	Pakistan	College student
Bhatt and Bhatt, 2015	244	India	College student
Yadav and Pathak, 2016	360	India	College student
Nam et al., 2017	542	United States	Consumers aged between 18 and 74
Choi and Johnson, 2019	284	United States	Adult
Mahesh, 2013	300	India	Consumers
Vazifehdoust et al., 2013	374	Iran	Consumers from the Guilan province in Iran
Hsu et al., 2017	300	Taiwan	Adult female
Gil and Jacob, 2018	469	India	Indian consumers who had prior experience in purchasing electrical and electronic products (EEP).
Nguyen and Nguyen, 2020	305	Vietnam	Vietnamese millennial consumers
Mei et al., 2012	230	Malaysia	A non-governmental organization - member of the activist group
Lao, 2014	909	China	Consumers around business centers
Sun et al., 2018	215	Hong Kong	Undergraduate students
Sun and Wang, 2019	654	China	Regular users of social media
Tan and Goh, 2018	304	Malaysia	Potential purchasers of green residential buildings over 21 years of age
Wu et al., 2015	305	Taiwan	Electric motorcycle users
Chaudhary, 2018	202	India	Students in higher education institutions
Ruangkanjanases et al., 2020	353	Taiwan	Participants with green products usage experience
Apipuchayakul and Vassanadumrongdee, 2020	288	Thailand	Customers living in Thailand that are over 18 years old with experience of purchasing lighting products
Wang X. et al., 2019	331	Tanzania	Middle-class
Wang X. et al., 2019	350	Kenya	Middle-class
Sheng et al., 2018	536	China	Customers of large stores
Smith and Paladino, 2010	157	Australia	Undergraduate students at an Australian University
Kang et al., 2013	701	United States, South Korea, and China	Participants were recruited from large universities in the United States, South Korea, and China
Pagiaslis and Krontalis, 2014	1695	Greek	18 years or older consumers
Karatu and Nik-Mat, 2015	440	Nigeria	Students attending university lectures
Mark and Law, 2015	457	Hong Kong	Respondents on the online survey
Achchuthan and Thirunavukkarasu, 2016	1325	Sri Lankan	Management undergraduates
Chen and Hung, 2016	406	Taiwan	Consumers with experience purchasing and using green products
Dhurup and Muposhi, 2016	386	South Africa	Generation Y consumers between the age of 19 and 36 years that were enrolled at two universities
Butt, 2017	574	Pakistan	18 years or older consumers are capable to make independent purchase decisions
Mai and Linh, 2017	303	Vietnam	Students from several universities in Hanoi
Mamun et al., 2018	380	Malaysia	Low-income household respondents who lived in coastal areas of Peninsular Malaysia
Arli et al., 2018	916	Indonesia	Student and nonstudent populations
Chaudhary and Bisai, 2018	202	India	Students
Taufique and Vaithianathan, 2018	175	India	Respondents on the online survey
Ali et al., 2019	400	Pakistan	Young consumers (18–30 years)
Teng and Wang, 2015	693	Taiwan	Food shoppers, customers at the age between 18 and 70
Al-Swidi et al., 2014	184	Pakistan	Academic staffs and students of two universities in southern Punjab
Varah et al., 2020	316	India	People in 20 shopping malls, 10 colleges and 5 residential areas in New Delhi

**TABLE 2:** Descriptive statistics.

Pairwise Relationship	Number of Studies	Range of correlations		Correlations		Range of sample sizes		Cumulative sample Size
					
		Lower	Upper	Significant	Non-significant	Lower	Upper	
GPV to GPI	6	0.378	0.698	5	1	258	536	2279
GPQ to GPI	5	0.198	0.534	5	0	215	469	1860
GPR to GPI	6	–0.460	–0.213	6	0	258	432	2016
PBC to GPI	27	0.024	0.730	19	8	184	916	11398
PCE to GPI	10	0.337	0.700	9	1	172	1325	4889
EK to GPI	9	0.238	0.456	7	2	230	1645	4322
EC to GPI	15	0.010	0.720	10	5	157	1695	7345
GT to GPI	7	0.086	0.680	4	3	236	693	2968
ATT to GPI	28	0.130	0.739	26	2	175	1325	13018
SN to GPI	27	0.081	0.757	18	9	157	1866	11958
Col to GPI	6	0.161	0.560	5	1	244	1866	3651

### Effect Sizes

The core objective of the meta-analysis is to calculate the effect values for comparative analysis. Effect values reflect the intensity and direction of the relations among variables. If, the effect value and variable relation are both positive, then the higher the effect value, the stronger and more stable the variable relation. This study adopted Hunter and Schmidt’s meta-analytic approach by using correlation coefficients to measure effect values (Hunter and Schmidt, 1990). Because of the different measurement methods across analyzed studies, it is necessary to treat the correlation coefficient as the standard effect value.

First, this study used the correlation coefficient *r* as the input effect size, adjust the effect value according to the following formula:

$$r^{{}^{\prime }}\,\frac{r}{\sqrt{\alpha_{\text{xx}}\,\alpha_{\text{yy}}}}$$

Where *r*^′^ is the correlation coefficient of each study after reliability correction, and *r* represents the correlation coefficient of each independent study, that is, the effect size; α_xx_ represents the reliability coefficient of the independent variable, and α_yy_ represents the reliability coefficient of the dependent variable. This step is to correct the attenuation deviation of correlation coefficient due to reliability defect of the scale. For the missing reliability values of some variables in few studies, we used the weighted average reliability of other similar research samples instead.

Second, obtaining the initial effect value using Fisher’s *Z* conversion of the effect size *r*^′^. The calculation formulas are as follows:

$${\text{Fisher}}^{{}^{\prime }}\,\text{s}\,Z_{\text{i}}=0.5\times \ln\,\frac{1+r^{{}^{\prime }}}{1-r^{{}^{\prime }}}$$

$$F\,i\,s\,h\,e\,r^{\prime }\,s\,Z_{+}=\frac{\sum n_{i}\,Z_{i}}{\sum n_{i}}$$

$$r_{z}=\frac{e^{2\times F\,i\,s\,h\,e\,r^{\prime }\,s\,Z_{+}}-1}{e^{2\times F\,i\,s\,h\,e\,r^{\prime }\,s\,Z_{+}}+1}$$

Where *r*^′^ is the correlation coefficient of each study after reliability correction, and *n*_*i*_ is the sample size corresponding to the influence value of *i*.

## Results

This study used Comprehensive Meta-Analysis 3.0 software. Meta-analysis is a statistical method for comprehensively and systematically analyzing many research results (Hedges, 1985). It has been widely used in medicine, psychology, pedagogy, management, economics, and other disciplines (Rosenthal and DiMatteo, 2001). This study primarily reports the meta-analysis results for publication bias, heterogeneity test, and combined effect value.

### Publication Bias Analysis

Publication bias is generally measured by the fail-safe *N* (Viechtbauer, 2007), which indicates that *N* unpublished articles must be added into the *K* sample of the current analysis to bring the final correlation coefficient or other significance levels below the critical value. The greater the difference between the *N*-value and *K*-value, the smaller the possibility of publication bias and the more stable the meta-analysis results. Borenstein takes “5k + 10” (where *k* is the number of independent effects included in the meta-analysis) as the threshold for publication bias: if the fail-safe *N* is greater than 5k + 10, there is no publication bias. As Table 3 shows, the fail-safe *N* is 10363 (*p* = 0.000, α = 0.05), which is well above the critical value of 280. As 10,363 unpublished studies would be needed to reverse this conclusion, there is no obvious publication bias problem in the analyzed studies.

**TABLE 3:** Publication bias test.

Classic fail-safe *N*	
*Z*-value for observed studies	122.51874
*P*-value for observed studies	0.00000
Alpha	0.05000
*Z* for Alpha	1.95996
Tails	2.0000
Number of observed studies	146.00000
Number of missing studies that would bring *p*-value to >alpha	10363.00000

**FIGURE 3:**
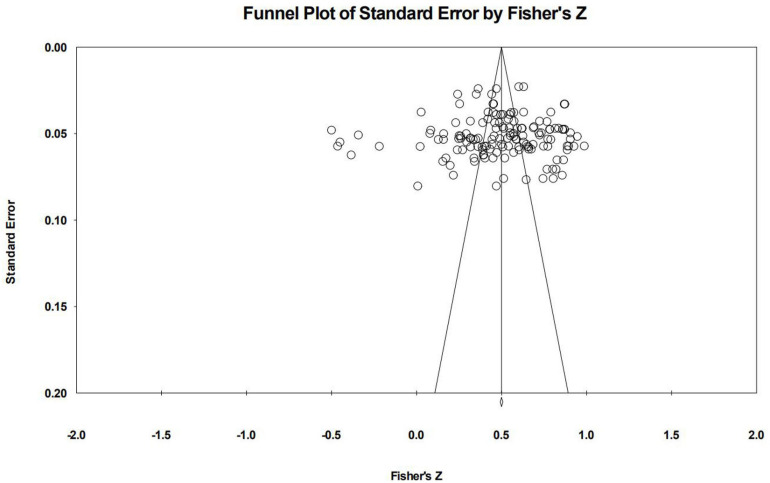
Funnel plot.

Figure 3 is a funnel plot of the selected samples in this study, which is the distribution of the effect value. The horizontal axis is the converted Fisher’s *Z* effect value, and the longitudinal axis is the standard error of the Fisher’s *Z* effect value. It can be seen from the figure that the funnel plot is usually funnel-shaped and symmetrically distributed, and the scattered points are mainly concentrated at the top of the funnel and near the average effect value, which indicates that the possibility of publishing bias in this study is very low.

### Heterogeneity Test

To evaluate the significant level of difference between different results, we need to conduct a heterogeneity test. The statistical results of heterogeneity are generally based on *Q*-value and I2 value. The *Q*-value was significant (*p* < 0.05), which indicates that there is heterogeneity between studies. *I*^2^ values of 25, 50, and 75%, respectively, correspond to low, middle, and high heterogeneity. If the distribution of the effect value is heterogeneous, a random-effects model is used; otherwise, a fixed-effects model is used. A fixed-effects model uses the differences within the study to calculate weight, assuming that all studies included in the meta-analysis use the same population and produce only one real effect value estimate. That is, the model considers that all possible factors affecting the effect value are the same, with any differences between studies coming only from negligible sampling errors. By contrast, a random-effects model uses intra- and inter-study differences to calculate weights, assuming that there are differences in the true effect values included in the meta-analysis and that the effect values depend on the subject characteristics, experimental methods, etc. This model also considers that the effect value calculation is influenced by between-study differences and within-study sampling errors. A random-effects model can also estimate the average value of the effective distribution and prevent underestimation of the weight of small-sample studies or overestimation of the weight of large-sample studies. Based on the results of the heterogeneity test, which are shown in Table 4, this study uses a random-effects model.

**TABLE 4:** Heterogeneity test.

Hypothesis	Pairwise relationship	*K*	*N*	Heterogeneity				Tau-squared			
				*Q*	df (Q)	*P*	I^2^	Tau squared	Standard error	Variance	Tau
H1	GPV to GPI	6	2279	55.890	5	0.000	91.054	0.027	0.019	0.000	0.165
H2	GPQ to GPI	5	1860	26.983	4	0.000	85.176	0.016	0.013	0.000	0.126
H3	GPR to GPI	6	2016	17.557	5	0.004	71.521	0.008	0.007	0.000	0.087
H4	PBC to GPI	27	11398	883.891	26	0.000	97.058	0.079	0.024	0.001	0.282
H5	PCE to GPI	10	4889	71.709	9	0.000	87.449	0.015	0.009	0.000	0.123
H6	EK to GPI	9	4322	19.392	8	0.013	58.747	0.003	0.003	0.000	0.058
H7	EC to GPI	15	7345	240.692	14	0.000	94.183	0.035	0.017	0.000	0.187
H8	GT to GPI	7	2968	171.243	6	0.000	96.496	0.067	0.042	0.002	0.258
H9	ATT to GPI	28	13018	573.880	27	0.000	95.295	0.044	0.014	0.000	0.211
H10	SN to GPI	27	11958	391.520	26	0.000	93.359	0.033	0.012	0.000	0.181
H11	Col to GPI	6	3651	128.090	5	0.000	96.096	0.049	0.037	0.001	0.222

### Sensitivity Analysis

Sensitivity analysis can also be understood as robustness analysis, which is an important method to evaluate the robustness and reliability of the combined results of meta-analysis. It is a common sensitivity analysis method to eliminate each included study one by one and then merge the effect quantity, change the exclusion criteria or eliminate a certain kind of literature before merging the effect quantity. Sensitivity analysis is to exclude meta analysis after low-quality research, or to include meta analysis after exclusion study. For example, after excluding a low quality study, the combined effect quantity is re-estimated and compared with the meta-analysis results before exclusion, and the influence degree and robustness of the study on the combined effect quantity are discussed. If the result does not change significantly after the exclusion, it means that the sensitivity is low and the result is more robust and credible; on the contrary, if there is a big difference or even the opposite conclusion after the exclusion, it means that the sensitivity is high and the robustness of the result is low. Based on the above theory, this study uses CMA software to perform the “one study removed” operation to check whether the effect size has changed. According to the results, it is found that the total effect value is 0.460, and the effect value between each relationship is distributed between 0.457 and 0.465. The results have not changed greatly, indicating that the sensitivity is low, and the results of this study are more robust and credible.

### Meta-Analytic Results

Table 5 shows the results of the meta-analysis of factors influencing green purchase intention. The results show a significant correlation between cognitive factors (*p* < 0.001) and green purchase intention. Green perceived value (*r* = 0.596) had the largest influence on green purchase intention. The effect size of perceived behavioral control (*r* = 0.499), perceived consumer effectiveness (*r* = 0.493), and green perceived quality (*r* = 0.462) each have medium correlations with green purchase intention. Green perceived risk (*r* = −0.373) had a negative effect on green purchase intention. Therefore, H1, H2, H3, H4, and H5 are supported.

**TABLE 5:** Meta-analysis results.

Hypothesis	Pairwise relationship	*K*	*N*	*R*	95% interval		Two-tail test	
					Lower limit	Upper limit	*Z*-value	*P*-value
H1	GPV to GPI	6	2279	0.596	0.499	0.678	9.680	0.000
H2	GPQ to GPI	5	1860	0.462	0.363	0.551	8.182	0.000
H3	GPR to GPI	6	2016	–0.373	–0.442	–0.300	–9.304	0.000
H4	PBC to GPI	27	11398	0.499	0.413	0.575	9.934	0.000
H5	PCE to GPI	10	4889	0.493	0.427	0.553	12.699	0.000
H6	EK to GPI	9	4322	0.335	0.289	0.379	13.470	0.000
H7	EC to GPI	15	7345	0.461	0.380	0.535	9.891	0.000
H8	GT to GPI	7	2968	0.530	0.376	0.656	5.935	0.000
H9	ATT to GPI	28	13018	0.535	0.475	0.589	14.564	0.000
H10	SN to GPI	27	11958	0.488	0.432	0.540	14.674	0.000
H11	Col to GPI	6	3651	0.352	0.184	0.500	3.960	0.005

*p < 0.001 indicates significant correlation; p < 0.05 indicates correlation.*

Regarding the relationship between consumer individual characteristics and green purchase intention, green trust (*r* = 0.530) and attitude (*r* = 0.535) were strongly correlated with green purchase intention, while environmental concern (*r* = 0.461) and environmental knowledge (*r* = 0.335) had a moderate correlation with this outcome variable. Therefore, H6, H7, H8, and H9 are supported.

For social factors, subjective norm (*r* = 0.488, *p* < 0.001) was significantly positively correlated with green purchase intention, and collectivism (*r* = 0.352, *p* < 0.05) was positively correlated with green purchase intention. Therefore, H10 and H11 are also supported.

## Discussion

This study used a meta-analysis to explore the influencing factors of green purchase intention. The results from 54 studies were analyzed, including 11 variables related to green purchase intention. The meta-analysis results show that green perceived value, attitude, and green trust each have a great positive impact on green purchase intention. Consumers are driven by value, often weighing the benefits and utility they gain when buying products (Kim et al., 2012). When consumers perceive the value to individuals and the environment from buying green products, they become more willing to buy green, which is consistent with previous findings (Chaudhary, 2018). Therefore, enterprises should increase the green perceived value as much as possible. Consumers with a positive attitude toward green products also have higher green purchase intention, which reinforces previous findings (Ruangkanjanases et al., 2020). The influence of green trust on green purchase intention reminds enterprises that they should convey to consumers the reliability and environmental protection that green products offer.

Perceived behavioral control, perceived consumer effectiveness, and subjective norm each have a moderate correlation with green purchase intention. The effect of perceived behavioral control mainly reflects consumers’ stronger willingness to buy when they are more confident in their purchasing ability, which has been identified in previous studies (Choi and Johnson, 2019). Therefore, enterprises should provide consumers with reliable information on the benefits of green products. Effective information is important for consumers’ decision-making process and can increase confidence in their purchasing ability. Similarly, if consumers are aware of the environmental protection resulting from individual green purchase behavior, their green purchase intention will increase. As consumer behavior is vulnerable to the influence of other people and group rules (Wang, 2014), relevant departments should strengthen the social norms of energy conservation and environmental protection.

Green perceived quality, environmental concern and environmental knowledge also have some influence on consumers’ green purchase intention. Consumers who pay more attention to the ecological environment and have relevant knowledge are more willing to buy green products (Choi and Johnson, 2019; Varah et al., 2020). Therefore, the government and enterprises should popularize relevant knowledge of environmental problems among consumers. Compared with other variables, collectivism is less significant. This shows that collectivism has less influence on green purchase intention.

Finally, green perceived risk has a negative impact on green purchase intention. The greater the risk consumers perceive in green products, the more reserved their attitude toward them and the lower their green purchase intention. Therefore, it is necessary for enterprises to minimize consumers’ perceived risk regarding green product purchases.

## Implications

Based on the results of the meta-analysis, this study can provide a reference for companies to formulate marketing policies. The results of this study show that consumers’ green perceived value, attitude and green trust are important indicators that affect consumers’ green purchase intention. If companies want to improve consumers’ green purchase intention, they should embed green perceived value, attitude and green trust in their long-term strategic planning. As the most important factors to influence green purchase intention, enterprises must improve green perceived value in order to improve consumers’ green purchase intention in the environmental era. Therefore, marketers should formulate marketing strategies to improve consumers’ perception of green value. Regarding attitudes, enterprise can use promotion and other methods to attract consumers’ attention to green products, provide consumers with more experience opportunities, establish a good image of the practicality of green products, and encourage more green attitudes. In addition, the government can also cultivate people’s green attitudes through social media and spread the benefits of green products to the public through various online channels. Companies should strengthen the environmental functions of green products and the environmental image of brands, and provide green products that meet consumer expectations to enhance consumer trust. In addition, enterprises can cultivate experienced retailers as effective and reliable information channels between consumers and manufacturers, salesmen should convey the environmental attributes and environmental protection effects of green products to consumers, so as to enhance consumers’ trust, and thus enhance their green purchase intention.

Moreover, we should also pay attention to the impact of perceived behavioral control, perceived consumer effectiveness and subjective norm on green purchase intention. When individuals are more confident in their purchasing ability, they are more likely to purchase products. In this perspective, enterprise should provide consumers with reliable information on the benefits of green products. Effective information is important to their decision-making process, which ensures that they have confidence in their ability to purchase green products. To enhance perceived consumer effectiveness, enterprises should transmit specific ideas to consumers through green labels, encourage them to participate in environmental protection, and clearly inform consumers how they contribute to environmental protection by buying green products. Policymakers can strengthen social norms of energy-saving behavior. For example, they could organize scale green environmental protection activities or use social media to disseminate norms encouraging more green purchasing behavior.

Besides, perceived risk also plays a role in explaining consumer behavior, because consumers are often motivated to reduce risk rather than maximize effects in the purchase process. Because green perceived risk reduces green purchase intention, marketers need to eliminate and reduce their perception of green risk in purchasing products.

## Conclusion

This study uses meta-analysis to analyze the factors that affect consumers’ green purchase intention based on previous studies. On the basis of the theory of consumer behavior, this study puts forward that the factors affecting consumers’ green purchase intention mainly include three categories: cognitive factors, consumer individual characteristics and social factors. Meanwhile, combine the expansion of TPB model and ABC theory, this study puts forward a theoretical framework to influence consumers’ green purchase intention. In previous studies, there is a conflict between some research results and a lack of consensus on the analysis of the influencing factors of consumers’ green purchase intention. This study uses the meta-analysis method to overcome the limitation of sampling error and sample size of a single study, and to solve the conflict results in the study of the influencing factors of consumers’ green purchase intention. The results show that, green perceived value, attitude, and green trust were most strongly positively correlated with green purchase intention, while perceived behavioral control, perceived consumer effectiveness, subjective norm, green perceived quality, and environmental concern were each moderately correlated with green purchase intention. Compared with other factors, environmental knowledge and collectivism had a relatively weak effect on green purchase intention, while the green perceived risk was negatively correlated with this outcome variable. According to the results, the assumptions involved in the study have been verified. This study’s findings enrich the theoretical basis for understanding consumers’ purchase intention for green products, provides new methods and ideas for exploring the influencing factors of green product purchase intention. Meanwhile, the results of this study can provide reference for enterprise marketing and government environmental protection propaganda. Enterprise marketers can make better use of the results of this study to formulate marketing strategies; government departments can publicize the advantages of green products and guide consumers to carry out environmental protection.

However, this paper has some shortcomings. First, due to incomplete information, the factors used for meta-analysis are limited to those for which sufficient data are available, some factors had to be excluded from the meta-analysis. Second, although heterogeneity in the sample was confirmed, we did not analyze the causes of heterogeneity. Third, there is no moderating effect test for the relationship in this study. In future studies, we should consider the variables with fewer data in this research, explore the influence of other factors on green purchase intention and the reasons for the heterogeneity.

## Data Availability Statement

The original contributions generated for this study are included in the article/supplementary material, further inquiries can be directed to the corresponding author.

## Author Contributions

WZ contributed to research design, reference searching, data coding, formal analysis, and manuscript writing. XL contributed to conceptualization, secondary data coding, and funding acquisition. MR contributed to data coding and manuscript revision. All authors contributed to the article and approved the submitted version.

## Conflict of Interest

The authors declare that the research was conducted in the absence of any commercial or financial relationships that could be construed as a potential conflict of interest.
